# Host–guest chemistry for tuning colloidal solubility, self-organization and photoconductivity of inorganic-capped nanocrystals

**DOI:** 10.1038/ncomms10142

**Published:** 2015-12-09

**Authors:** Maryna I. Bodnarchuk, Sergii Yakunin, Laura Piveteau, Maksym V. Kovalenko

**Affiliations:** 1 Laboratory of Inorganic Chemistry, Department of Chemistry and Applied Biosciences, ETH Zürich, Vladimir Prelog Weg 1, CH-8093 Zürich, Switzerland; 2 Laboratory for Thin Films and Photovoltaics, Empa—Swiss Federal Laboratories for Materials Science and Technology, Überlandstrasse 129, CH-8600 Dübendorf, Switzerland

## Abstract

Colloidal inorganic nanocrystals (NCs), functionalized with inorganic capping ligands, such as metal chalcogenide complexes (MCCs), have recently emerged as versatile optoelectronic materials. As-prepared, highly charged MCC-capped NCs are dispersible only in highly polar solvents, and lack the ability to form long-range ordered NC superlattices. Here we report a simple and general methodology, based on host–guest coordination of MCC-capped NCs with macrocyclic ethers (crown ethers and cryptands), enabling the solubilization of inorganic-capped NCs in solvents of any polarity and improving the ability to form NC superlattices. The corona of organic molecules can also serve as a convenient knob for the fine adjustment of charge transport and photoconductivity in films of NCs. In particular, high-infrared-photon detectivities of up to 3.3 × 10^11^ Jones with a fast response (3 dB cut-off at 3 kHz) at the wavelength of 1,200 nm were obtained with films of PbS/K_3_AsS_4_/decyl-18-crown-6 NCs.

## Abstract (web summary)

The high polarity of colloidal inorganic-ligand-functionalized nanocrystals can be problematic for their processing, limiting their optoelectronic applications. Here, by complexation with macrocycles, the authors enabled broad amphiphilicity of such nanocrystals and processing from a variety of solvents.

## Graphical abstract

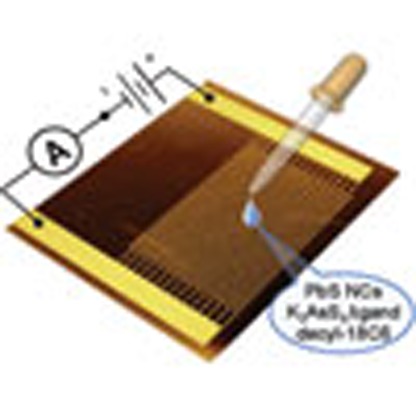

New strategies for the programmable, scalable and experimentally facile bottom-up design of solid-state materials from chemically synthesized nanocrystals (NCs) are becoming increasingly important because of their promising applications in electronic and optoelectronic devices^1^,^2^,^3^,^4^,^5^,^6^. In this regard, NC surface chemistry has become a major bottleneck and, at the same time, a major enabler due to its crucial influence on nearly all of the physical and chemical properties of NCs^5^,^7^. Bulky and electrically insulating organic capping ligands (surfactants) enable the formation of monodisperse colloidal NCs. In the direction towards subsequent applications of NCs in solid-state devices, the last several years have seen a surge of reports showing the great potential of small, electrically conductive inorganic capping ligands, such as metal chalcogenide complexes (MCCs, for example, Sn_2_S_6_^4−^, SnTe_4_^4−^, Cu_7_S_4_^−^, In_2_Se_4_^2−^ and AsS_4_^3−^)^8^,^9^,^10^,^11^,^12^,^13^,^14^,^15^,^16^,^17^,^18^,^19^,^20^ and other ionic inorganic moieties (for example, halides, pseuodohalides, metal-free chalcogenido, hydroxyl and amido ions, oxo- and polyoxometallates, and halometalates)^12^,^17^,^21^,^22^,^23^,^24^,^25^,^26^. All of these ligands have been used to displace initial organic capping ligands, forming stable colloidal dispersions. Another powerful strategy, applicable to metal oxide and chalcogenide NCs, is the formation of cationic and naked, but colloidally stable NCs upon controlled stripping of the strongly bound native organic ligands with the simultaneous coordination of metal cations on the NC surface with weakly nucleophilic ligands^26^,^27^,^28^. Such all-inorganic NC surface chemistries have enabled major advances in the design of solid-state NC devices: high electronic mobilities in densely packed NC films (5–35 cm^2^ V^−1^ s^−1^)^12^,^16^,^22^,^29^,^30^,^31^,^32^,^33^, the integration of highly luminescent PbS/CdS NCs into fully inorganic infrared transparent matrices^13^, efficient photoconductivity in the visible and near-infrared regions with detectivities reaching 10^13^ Jones^34^, improved electrocatalytic properties of CdSe NCs (hydrogen evolution)^26^ and enhanced electronic transport in NC-based Li-ion batteries^25^. Inorganic-capped NCs also served as fully inorganic inks and precursors for CuInS_2_, Cu(In_1–*x*_Ga_*x*_)Se_2_, Cu_2_ZnSn(S,Se)_4_ and PbS phases as thin-film absorbers for photovoltaics^11^,^35^,^36^, CdSe thin films with exceptionally high electron mobilities of up to 200 cm^2^ V^−1^ s^−1^ (ref. 37), ionically conductive composites of Ag_2_S NCs embedded into a GeS_2_ matrix^38^, Sb_2_Te_3_-based nanocomposites for thermoelectrics^20^,^39^, and NC-in-glass windows with electrochemically tunable transmittance^40^,^41^. MCCs have been employed as capping ligands in the majority of these reported examples of solid-state devices/materials. The versatility of MCCs as ligands is rooted in their exceptionally strong binding affinity to NC surfaces, including commonly studied NCs of metals (Au and Pd) and semiconductors (CdSe, PbS, InAs, InP and so on). Ligand exchange with MCCs proceeds quantitatively to completion in most cases and leads to the phase transfer of NCs from nonpolar to highly polar solvents, such as hydrazine or *N*-methylformamide (MFA), forming concentrated, long-term stable colloidal solutions. In such highly polar media (*ɛ*=40–190), the strong adsorption of anions combined with the dissociation of cations leads to efficient electrostatic colloidal stabilization. Straightforward solution-based processing with common techniques such as spin- or spray-coating has led to the aforementioned success of inorganic-capped NCs in solid-state devices. At the same time, MCC-capped NCs lack the ability to disperse in moderately polar and apolar solvents and/or to mix with molecules of various polarities, and exhibit limited ability to form regular and ordered NC solids.

Herein we demonstrate a simple approach to continuously tune the surface functionality of MCC-capped NCs from highly polar to intermediately and finally rather nonpolar. Consequently, the range of solvents and other media in which inorganic-capped NCs can be dispersed is greatly extended, and includes, for instance, common volatile organic solvents such acetonitrile, acetone and toluene. This is achieved here by the host–guest coordination of MCC-capped NCs with macrocyclic ethers—crown ethers^42^ and cryptands^43^—as host molecules for the alkali metal counter ions of MCCs, as depicted in Fig. 1. This approach turns the cationic side of the all-inorganic surface capping into an important factor for adjusting chemical and physical properties of NCs in the colloidal state and in thin films. Since the macrocycle molecule is neutral, and the stability of the resulting complex is highly solvent dependent, the macrocycle can be removed from the NC film by merely dipping it into the ‘wrong' solvent. Instead of being removed, this organic corona can also be used for fine-tuning of the charge transport, as demonstrated here for the photoconductive properties of PbS NCs. In films of PbS/K_3_AsS_4_/decyl-18-crown-6 NCs, high-infrared-photon detectivities of up to 3.3 × 10^11^ Jones with a fast response (3 dB cut-off at 3 kHz) at the wavelength of 1,200 nm were obtained. On the other hand, films of crown-free PbS/K_3_AsS_4_ NCs are excessively conductive and therefore not suitable for observing efficient photocurrent generation, while the initial oleate-capped PbS NCs are fully insulating, precluding photocurrent measurements.

## Results

### Host–guest approach to tune the solubility of NCs

**Figure 1:**
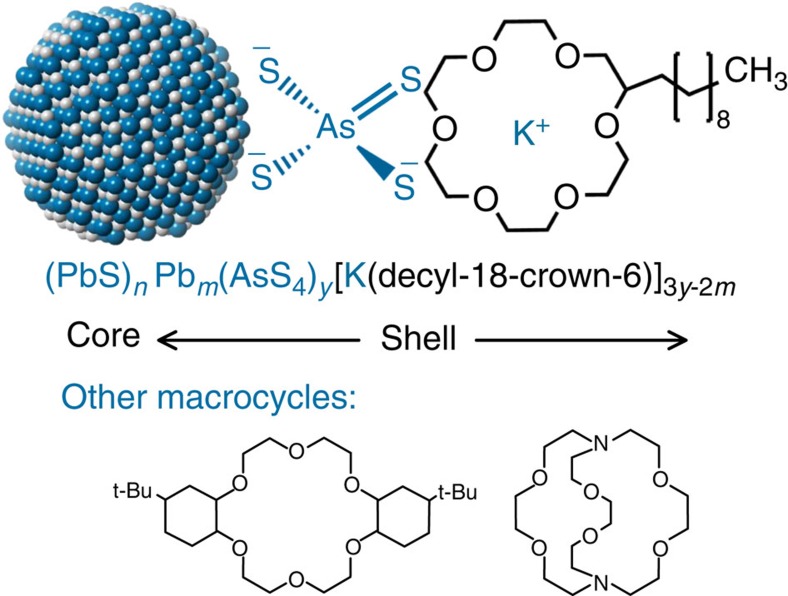
Schematic of the complexation of inorganic-capped colloidal NCs with crown ethers or cryptands. The binding of a neutral macrocyclic polyether, decyl-18-crown-6, onto the surface of a PbS NC capped with K_3_AsS_4_ occurs via dipole–ion interactions. The entire assembly is held together by both the strong ion pairing and moderately strong host–guest interactions. The corona of macrocyclic molecules governs the dispersibility of such NCs in various solvents.

Multiple etheral oxygen atoms are capable of coordinating metal cations. Owing to such dipole–ion interactions and geometric effects, cyclic (crown ethers) and polycyclic (cryptands) polyether molecules are known to selectively coordinate very small and hard alkali metal cations (Li^+^, Na^+^ and K^+^), enclosing them within the interior of donut-shaped rings (crowns) or cages (cryptands). Since the pioneering work in the 1960s on crown ethers by Pedersen^42^ and on cryptands by Lehn^43^, these host molecules have been used in organic and inorganic chemistry as phase transfer and solubilizing agents (especially for isolating peculiar compounds such as electrides, alkalides and Zintl phases), as phase transfer catalysts, or as discerning host molecules in ion-selective electrodes^44^. ‘Host–guest' coordination has launched modern supramolecular chemistry and still stirs the imaginations of many scientists today, towards boundless molecular structures and applications^45^,^46^. Just as in Pedersen's classic experiment demonstrating the utility of crown ethers for the solubilization of KMnO_4_ in benzene, leading to so-called ‘purple' benzene due to the formation of lipophilic [K(18-crown-6)]MnO_4_, we demonstrate in this work a practical route to the solubilization of MCC-capped colloidal NCs in moderately polar or fully nonpolar solvents using similar approach. For one selected system, PbS NCs/K_3_AsS_4_ ligand/decyl-18-crown-6 macrocycle (Fig. 1), we present a detailed characterization and discuss the key aspects of this supramolecular approach and its effect on solubility in various solvents, self-assembly into long-range ordered superlattices and (photo)conductive properties. We also briefly discuss other NC–ligand–macrocycle combinations tested in the course of this work.

**Figure 2:**
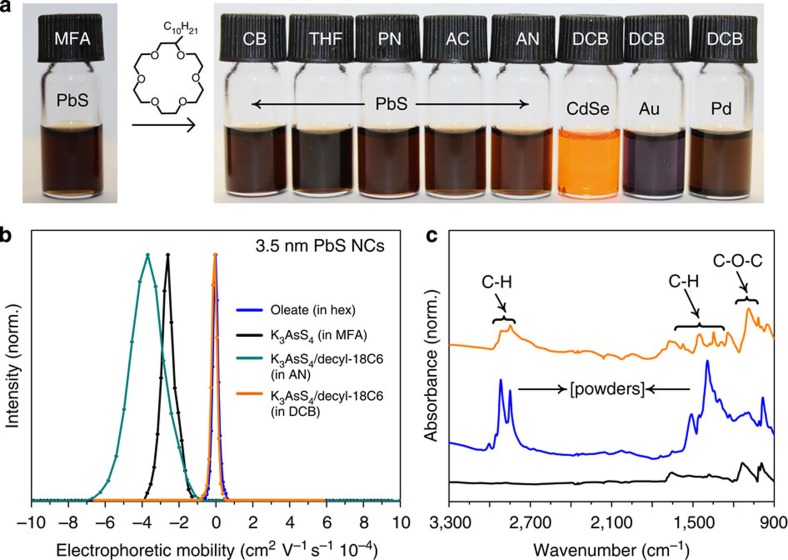
Dispersibility tuning of inorganic-capped colloidal NCs using a host–guest supramolecular approach. (**a**) Stable colloidal solutions of various metallic and semiconductor K_3_AsS_4_-capped NCs in common polar and nonpolar solvents, obtained by solubilizing with decyl-18C6; (**b**) electrophoretic mobility measurements for oleate-, K_3_AsS_4_−, and K_3_AsS_4_/decyl-18C6-capped PbS NCs, and (**c**) the corresponding FTIR spectra of powdered samples. Solvents: MFA, *N*-methylformamide; CB, chlorobenzene; THF, tetrahydrofurane; PN, pyridine; AC, acetone; AN, acetonitrile; DCB, dichlorobenzene.

The initial carboxylate-capped 3.5–10 nm PbS NCs consist of a stoichiometric PbS core covered with a Pb-oleate monolayer^47^. After ligand exchange, elemental analysis of 4 nm PbS NCs using Rutherford backscattering spectrometry (RBS) indicates that the Pb-rich stoichiometry is retained, confirming that oleate anions are stripped off the surface and replaced with polydentate AsS_4_^3−^ ions. The remaining charge balance is provided by K^+^ cations. Such NCs are only dispersible in highly polar solvents (for example, MFA with a static dielectric constant that is a factor of 2 higher than that of water) since efficient electrostatic dissociation of K^+^ counter ions is required. Here we use these K^+^ cations to attach charge-neutral macrocycles via weak host– (macrocycle) guest (K^+^) interactions, as depicted in Fig. 1. As a result, the surface chemistry of the NCs becomes rather amphiphilic, that is, making them dispersible in both polar and nonpolar media, due to electrostatic and steric effects. The relative contributions of electrostatics and steric interactions between the NCs and with the solvent are dependent on the selection of macrocycle and solvent, as discussed below. In a typical procedure, inorganic-capped NCs are precipitated from MFA by adding acetonitrile or an acetonitrile/toluene mixture, separated by centrifuging, and redispersed in acetonitrile containing decyl-18-crown-6 (decyl-18C6, 0.1 M), instantly forming a colloidally stable solution. Acetonitrile is chosen as a solvent since it exhibits high stability constants of host–guest complexes, reported to be among the highest for all tested macrocycles. It is important to note that inorganic-capped NCs cannot be dispersed in acetonitrile in the absence of macrocycles. To disperse ‘crowned' NCs in other solvents (Fig. 2a), acetonitrile is simply evaporated and the desired solvent is added (chlorobenzene, dichlorobenzene, acetone, pyridine, tetrahydrofurane and so on). The proposed methodology of preparing supramolecular assemblies from inorganic-capped NCs and macrocycle molecules is very versatile. In addition to PbS, we also successfully tested NCs of CdSe (3 nm), metals (Au and Pd, 3–4 nm) and core–shell PbS/CdS and CdSe/CdS NCs. These NCs were combined with either K_3_AsS_4_ ligands or with K_4_SnS_4_ and K_4_GeS_4_. In addition to decyl-18C6, a variety of commercially available macrocycles were also tested: dicyclohexano-18-crown-6 (DCH-18C6, from Merck), dibenzo-18-crown-6 (DB-18C6, from Merck), 4′,4″(5″)-di-tertbutyldicyclohexano-18-crown-6 (DTB-DCH-18C6, from Aldrich, see Fig. 1), 1,10-didecyl-1,10-diaza-18-crown-6 (DD-diaza-18C6, from Aldrich), 4,7,13,16,21,24-hexaoxa-1,10-diazabicyclo[8.8.8]hexacosane (cryptand C222, from ABCR, see Fig. 1) and 5,6-benzo-4,7,13,16,21,24-hexaoxa-1,10-diazabicyclo[8.8.8]hexacos-5-ene (benzyl-C222, from Aldrich). It is worth noting that a variety of alkylated lipophilic derivatives of cryptands have been reported in the literature and may be of great interest as well, such as C_10_- and C_14_-grafted C222 cryptands^48^. All tested macrocycles allow the transfer of NCs into solvents of lower polarity (*ɛ*=2–40) than the solvents in which pristine inorganic-capped NCs can initially be dispersed (*ɛ*=40–191, the highest for MFA and *N*-methylacetamide). As expected, access to rather nonpolar solvents such as CB and DCB is provided only by macrocycles containing aliphatic and aromatic side groups, with DTB-DCH-18C6, and decyl-18C6 being the most successful among the examples tested herein.

Electrophoretic mobility measurements (Fig. 2b) indicate that with the addition of decyl-18-crown-6 onto the surface of the NCs, followed by redispersion in a solvent of lower polarity (*ɛ*<10, for example, chlorobenzene, toluene and so on), the effective surface charge (measured as zeta-potential) is reduced due to ion pairing between the ligand anions and counter cations. In addition, a steric component is provided by the decyl chain. In a solvent of medium polarity, such as acetonitrile (acetonitrile, *ɛ*=37.5), no ion pairing occurs and instead a highly negative surface potential is observed; and the role of the macrocycle in this case is to aid the electrostatic dissociation of the cation from the NC surface. In fact, corresponding zeta-potentials of PbS/K_3_AsS_4_ NCs in MFA and PbS/K_3_AsS_4_/decyl-18C6 NCs in acetonitrile are similarly high: ca. −50 V (Supplementary Fig. 1). These examples illustrate the tunable amphiphilicity provided by the macrocycle and its dependence upon the solvent. The true colloidal nature of the obtained solutions is confirmed by dynamic light scattering measurements, showing single-particle populations (Supplementary Fig. 2) and the absence of any macroscopic aggregates. The slightly larger hydrodynamic radius of PbS/K_3_AsS_4_/decyl-18C6 compared to initial and ligand-exchanged colloids indicate the contribution from the corona of macrocycles. Fourier-transform infrared (FTIR) spectra of powdered samples verify the removal of the oleate ligand and, after ligand exchange, the attachment of the macrocycle forming PbS/K_3_AsS_4_/decyl-18C6 (Fig. 2c). Elemental analysis by the RBS (Supplementary Table 1) indicates that the K_3_AsS_4_ capping layer remains intact (that is, does not desorb from the NC surface) throughout the subsequent manipulations with macrocycles and solvents. A direct evidence for the coordination of macrocycle to the NC surface is provided by the ^1^H nuclear magnetic resonance (NMR) spectroscopy (Supplementary Figs 3 and 4, and related discussion). Binding of the organic moieties to the NC surface results in significant broadening of solution NMR signals due to slower tumbling and, therefore, slower relaxation of molecule, smearing out the identity of the NMR peaks. Etching of the NCs by a dilute acid liberates the macrocycle and restores sharp features in the NMR signal.

As a result of addition of macrocycles, stable NC dispersions can be obtained in solvents of medium polarity (acetonitrile, acetone, pyridine and so on) which are traditionally inaccessible for purely charge- or purely sterically stabilized colloids. Previously, the solubilization of MCC-capped NCs in highly nonpolar solvents (toluene and chloroform) has been achieved via the exchange of alkali metal cations with bulky alkylammonium cations, such as didodecyldimethylammonium bromide (DDAB)^9^; however, dispersibility in solvents of medium polarity was not achieved. The resulting DDA–inorganic ligand–NC assembly features a strongly bound DDA–ligand ionic pair that can be dissociated only by highly invasive chemical means such as solid-state cation exchange or thermal decomposition. The host–guest supramolecular approach presented here does not establish any direct, strong interaction between the macrocycle and inorganic ligand and is much easier to dissociate when needed.

### Self-organization of ‘crowned' nanocrystals

**Figure 3:**
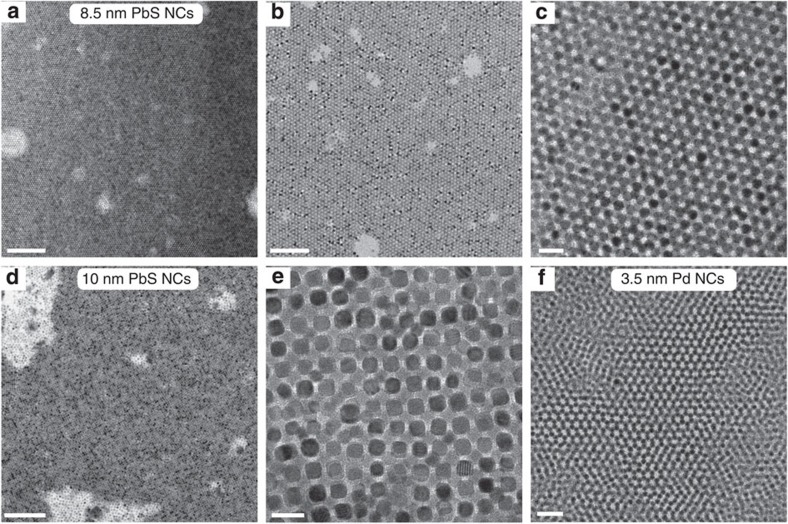
Host–guest chemistry enables self-organization of inorganic-capped NCs into long-range ordered superlattices. TEM images of superlattices prepared from K_3_AsS_4_/decyl-18-crown-6-capped (**a**–**c**) 8.5 nm PbS NCs, (**d**,**e**) 10 nm PbS NCs and (**f**) 3.5 nm Pd NCs. Scale bars are 200 nm (**a**), 100 nm (**b**), 20 nm (**c**), 200 nm (**d**), 20 nm (**e**), 20 nm (**f**).

An important problem in NC self-assembly is balancing the entropic factors (symmetry and packing density) with specific interactions such as Coulomb interactions of surface charges, dipole–dipole interactions and van der Waals forces. Colloids of inorganic-capped NCs are stabilized electrostatically, for example, through the formation of an electric double layer. Because of strong long-range electrostatic repulsion, such NCs rarely pack into long-range ordered structures, presumably because of a suppressed ability to carry out dynamic structural reorganization at short distances at a late stage of solvent evaporation. In contrast, short-range steric stabilization of organically passivated NCs allows the facile growth of various long-range ordered superlattices from nonpolar organic solvents upon simple drying^49^,^50^. Both entropy and weak short-range energetic interactions (dispersion forces and dipole–dipole interactions) govern the assembly of colloidal NCs^51^,^52^,^53^, contributing to the change in Gibbs free energy of the system: d*G*=d*H*−*T*d*S*. The gain in free volume entropy upon ordering is greater than the decrease in configurational entropy, thus providing a net positive change to the system's entropy and driving assembly into close-packed structures. In full agreement with these arguments, a transmission electron microscopy (TEM) study of the NC films obtained from all tested solvents shows that indeed only the charge-neutral, sterically stabilized NCs in nonpolar solvents form long-range ordered superlattices upon solvent evaporation (Fig. 3). NCs smaller than 10 nm (PbS and Pd, Fig. 3a–c,f) tend to form a hexagonal close-packed lattice, while larger, cubic-shaped PbS NCs (Fig. 3d,e) form a body-centred cubic lattice.

### Photoconductive properties of ‘crowned' PbS NCs

In the past several years, PbS NCs have become exceptional materials for optoelectronic applications, showing promise for photovoltaics^3^,^6^ as well as for photon detection in the visible and infrared spectral regions^2^,^54^,^55^,^56^,^57^. In photoconductive devices, both an extremely high light responsivity (outperforming commercial devices)^2^ and a high speed of response to light^56^,^58^ have been reported. A general trend for most photoconductors is that higher sensitivities are accompanied by slower response (that is, a smaller bandwidth *B*). This is consistent with the key role played by charge trapping states, controlled mainly by the surface chemistry, that are responsible for photoconductive gain effects in photoactive nanocrystalline materials^59^. The photoconductive gain (*G*) is the ratio between de-trapping and transit times of localized and mobile carriers, respectively. Thus, long-lived traps lead to multiple passes of mobile carriers through the device, increasing *G*^1^. De-trapping time is known to increase with the depth of the trap states, with respect to the bottom of the conduction band (for electrons) or top of the valence band (for holes), in both bulk materials^60^ and in NCs^58^. For PbS NCs, an empirical figure of merit *G* × *B* can be used. When *G* and *B* are measured under similar, ideally low light intensity, the *G* × *B* product for optimized PbS QD photodetectors should be in the range of 10^4^–10^5^ Hz (refs 57, 58). Hence for the rational engineering of photodetector characteristics (sensitivity, detectivity and response time) control over the depth and density of trap states is critical^57^. Judicious choice of inorganic capping ligands enables control over the trapping/de-trapping dynamics. Here we show that also the organic corona of macrocycles can be used to tune *G* and *B*, while preserving the expected *G* × *B* product. The observations described below can be attributed to the combined effect of the improved short-to-medium range order in the arrangement of the NCs within the film, and adjustments of the mean interparticle spacing.

The resulting conductive and photoconductive properties of NC–ligand–macrocycle films are governed by all three adjustable components: composition of a NC, selection of inorganic ligand and macrocycle. Films of oleate-capped 3 nm PbS NCs are highly insulating, with conductivities typically below 10^−11^ S cm^−1^. K_3_AsS_4_/decyl-18C6 or K_3_AsS_4_/C222 capping renders PbS NC films 10^4^-fold more conductive: 0.2 and 0.1 μS cm^−1^, respectively. Fully inorganic capping with only K_3_AsS_4_ leads to even higher conductivities of up to 25 μS cm^−1^, confirming the prevailing role of the ligand capping for charge transport. In the case of equally sized metallic Pd NCs, a further increase in conductivity is observed with both K_3_AsS_4_/decyl-18C6 capping and with K_3_AsS_4_ as the sole ligand (20 and ∼100 S cm^−1^, respectively). This demonstrates the significance of the NC core, presumably contributing mainly to the overall carrier density of the composite, with metals offering the highest number of mobile carriers. AsS_4_^3−^ is electronically insulating by itself, but owing to its small size, a thin capping layer of K_3_AsS_4_ mediates tunnelling and charge hopping within the NC film. While these general observations shed important insight on the conductive properties of NC–ligand–macrocycle films, charge transport in NC solids is a matter of high complexity and for more detail the reader is referred elsewhere^1^,^6^,^7^,^61^.

**Figure 4:**
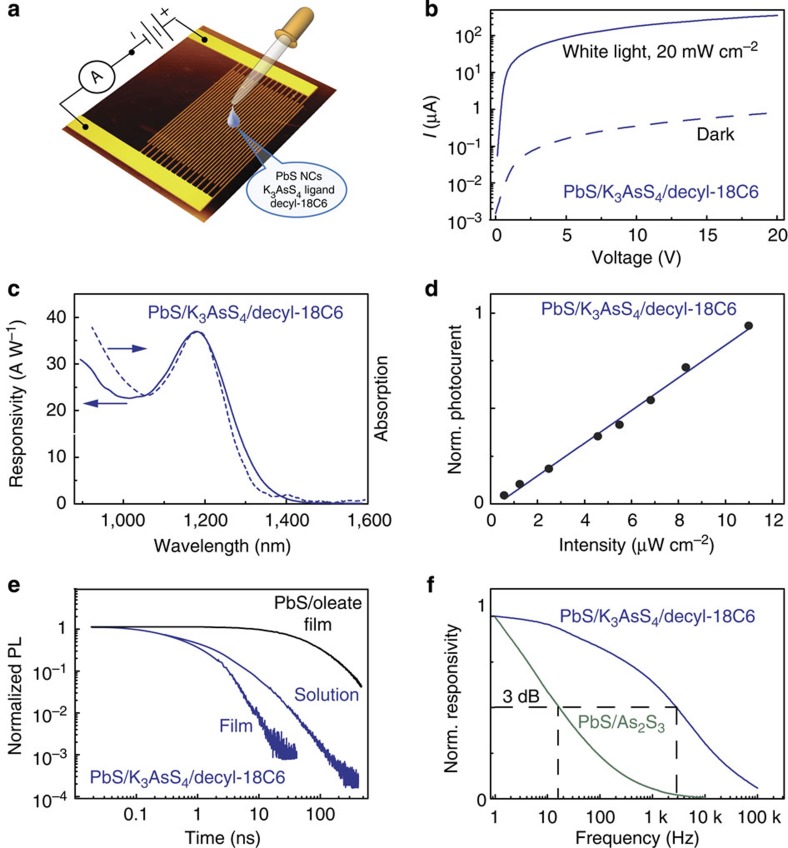
Photoconductive properties of ‘crowned' PbS NCs. (**a**) Schematic of a solution processed photodetector; the pseudo-three-dimensional image of a glass substrate with interdigitated gold electrodes was rendered with Gwyddion software from a microphotograph of a real device; (**b**) current–voltage characteristics for ∼3 nm PbS NCs capped with K_3_AsS_4_/decyl-18C6 in darkness (dashed line) and under white-light illumination of 20 mW cm^−2^ (solid line); (**c**) responsivity spectrum of the same film; (**d**) intensity dependence of the photocurrent during illumination at the excitonic peak (*λ*≈1,200 nm); (**e**) time-resolved photoluminescence decays from films and solutions before and after ligand-exchange; (**f**) frequency dependence of the photocurrent for PbS NCs capped with K_3_AsS_4_/DC (blue) and PbS NCs with an amorphous As_2_S_3_ capping layer (green). Norm., normalized.

For photoconductivity experiments, PbS NCs/K_3_AsS_4_/decyl-18C6 colloids were drop casted over interdigitated electrodes with 10 μm gaps and a total channel length of 43 mm (Fig. 4a). Upon illumination, PbS NC films exhibit high photocurrent, which is 100–1,000 times higher than the current in darkness (Fig. 4b). The responsivity spectrum (Fig. 4c) closely matches the near-infrared absorption spectrum, with a pronounced excitonic peak. The responsivity exhibits a highly linear dependence on the incident light intensity (Fig. 4d). The responsivity value of ∼40 A W^−1^ can be estimated at the excitonic peak of 1,200 nm, yielding *G* of at least 40. With the noise equivalent power of ∼2 × 10^−13^ W Hz^−½^, a high detectivity of 3.3 × 10^11^ Jones is estimated at a wavelength of 1,200 nm. A value of *G*≥1 indicates the localization of one carrier type in trap states. Sulfidoarsenate and other chalcogenide ligands were previously reported to act as hole traps^57^. For instance, highly electronically passivated oleate-capped PbS NCs initially exhibit high-photoluminescence quantum yields of up to 40% and long radiative lifetimes on the order of hundreds of nanoseconds (Fig. 4e). Upon exchange with K_3_AsS_4_, photoluminescence quantum yields decrease by 1–2 orders of magnitude and average photoluminescence lifetimes are as short as one to several nanoseconds, in both solutions and films, with and without macrocycles attached (Supplementary Fig. 5). Hence, we conclude that trapping of holes does play an important role in this study as well and is a primary charge carrier trapping mechanism.

The de-trapping time is revealed by the frequency dependence of the photoresponse (Fig. 4f) and, as discussed above, provides a trade-off between the speed and sensitivity of the detector. A 3 dB cut-off is located at ∼3 kHz. For comparison, we have recently reported highly sensitive all-inorganic PbS/As_2_S_3_ photodetectors with higher sensitivities (200 A W^−1^) and detectivities (2 × 10^13^ Jones) at the same wavelengths, but with a sacrifice of the response times; correspondingly, these exhibited a slow frequency response with a 3 dB value of just 10 Hz. *G* × *B* products of both detectors are 1–2 × 10^5^ Hz. The much faster photoresponse of PbS/K_3_AsS_4_/decyl-18C6 films can be explained by the fact that discrete AsS_4_^3−^ has a wider gap between the highest occupied molecular orbital and lowest unoccupied molecular orbital (HOMO-LUMO gap) and does not introduce as many nor such deep trap states as the much narrower gap amorphous As_2_S_3_ semiconducting capping layer generated by decomposing (NH_4_)_3_AsS_3_ ligands in our previous work^57^.

## Discussion

This study shows that weak (non-covalent and non-ionic) structure-specific interactions of high selectivity can lead to very useful adjustments of the chemical and physical properties of colloidal inorganic NCs and their solids. NC–ligand–crown and NC–ligand–cryptand assemblies exhibit broadly tunable dispersibility in various solvents, behaving as amphiphilic moieties due to both steric and electrostatic contributions to the interparticle interactions. This approach also assists in self-organization of NCs by switching the repulsive forces responsible for colloidal stabilization of inorganically capped NCs from long-range electrostatic to short-range steric. Besides acting as surface-active reagents, macrocycles also have a profound effect on the electronic properties of NC solids. For instance, high responsivities of up to 40 A W^−1^ and a high speed of operation (bandwidth of 3 kHz) can be demonstrated. While here we only utilized commercially available crown ethers, cryptands and their derivatives, a variety of other macrocycles may also potentially be used for such purposes. Examples of the latter include calixarenes, acyclic molecules such as oligo-ethylene-glycols, and many natural molecules (for example, valinomycin) that exhibit a known alkali metal-specific binding.

## Methods

### Synthesis of NCs

PbS NCs (3–11 nm) capped with oleic acid were synthesized from lead oleate and bis(trimethylsilyl)sulfide as reported elsewhere^62^. The size of the PbS NCs was controlled by varying the molar ratio of oleic acid to octadecene. Wurtzite-phase CdSe NCs (3 nm) were synthesized according to the procedure reported by the Bawendi group^63^. OAm-capped Au NCs (3.8 nm)^64^ and Pd NCs (4 nm)^65^ were also synthesized as reported elsewhere.

### Ligand exchange with K_3_AsS_4_

K_3_AsS_4_ was prepared by mixing stoichiometric quantities of As_2_S_5_ and K_2_S in water for 10 h and isolated by adding acetonitrile, followed by drying. Freshly prepared K_3_AsS_4_ (5 mg) was dissolved in 1 ml of MFA and mixed with 5 mg of PbS NCs dispersed in 1 ml of hexane. The two-phase system was stirred vigorously for 1 h. The NCs in MFA were rinsed three times with 5–6 ml of hexane and filtered through a 0.2 μm PTFE filter.

### Host–guest chemistry with macrocycles

NCs in MFA (100 μl, 5 mg ml^−1^) were precipitated by the addition of 0.4–1 ml of toluene or toluene/acetonitrile mixture, followed by centrifugation. Then, 40–100 μl of the solution of macrocyclic polyethers in acetonitrile (0.05–0.1 M) were added to redisperse the NCs. Acetonitrile was removed by drying under vacuum. The NCs were then redispersed in DCB. To remove excess macrocycles, the NCs were precipitated with 0.1–0.5 ml of ethanol or an ethanol/hexane mixture. Thus obtained NCs were fully dispersible in nonpolar solvents such as CB, DCB, THF, PN and so on.

### Preparation of NC superlattices

A solution of NC-macrocycle assemblies in CB or DCB (20–30 μl) with a NC concentration of ∼10^14^–10^15^ NCs per ml was placed onto a carbon-coated TEM grid and dried at 50–55 °C in the closed chamber under nitrogen for several hours.

### Characterization

TEM images were recorded using a JEOL JEM-2200FS microscope operated at 200 kV. Electrophoretic mobility and dynamic light scattering measurements were performed using a Zetasizer Nano-ZS instrument (Malvern Instruments, Inc.). Colloidal solutions were tested using a dip-cell setup with Pd electrodes. FTIR spectra were measured using a Thermo Scientific Nicolet iS5 FTIR spectrometer. RBS was performed at the ETH Laboratory of Ion Beam Physics. Measurements were conducted using a 2 MeV ^4^He beam and a silicon PIN diode detector under an angle of 168°. The collected RBS data were analysed using simulations by the RUMP code^66^. NMR spectra were acquired on a Bruker 11.7 T spectrometer equipped with PABBO gradient field three channel liquid-state probe head for 5 mm NMR tubes. Experiments were performed at room temperature in non-spinning mode while locking the deuterium signal of the CD_3_CN. The 90° pulse and the recovery delay for ^1^H was 11.40 μs and 5 s, respectively 9.50 μs and 2 s for ^13^C. One-dimensional spectra were acquired with a single pulse sequence acquiring 1 scan for ^1^H and 1,024 scans for ^13^C. Chemical shifts were referenced to Si(CH_3_)_4_ as an external standard.

Photoconductivity measurements were performed with illumination from a tungsten lamp dispersed by an Acton SP2150 (Roper Scientific) spectrograph/monochromator. For measurements of the responsivity spectra, the light was modulated by a mechanical chopper at a frequency of 33 Hz. The sample was biased using a Keithley 236 SMU by 10–20 V, limited to obtain stable dark currents in the range of 1–2 μA. The signal, amplitude and phase were measured across a series resistant by a Stanford Research 830 lock-in amplifier. The light intensity was controlled by a calibrated power detector (UM9B-BL, Gentec-EO). Current–voltage (*I*–*V*) characteristics were also measured by the Keithley 236 SMU. Frequency dependencies were obtained with a load resistance of 1,000 Ω, under pulsed and focused illumination from an LED emitting at a wavelength of 1,200 nm.

Time-resolved photoluminescence traces in the infrared region were obtained by a Time-Correlated Single Photon Counting (TCSPC) system based on an InGaAs TE-cooled single-photon avalanche photodiode (ID Quantique) with 200 ps time resolution, adjusted for 10% quantum efficiency, using a SPC-130-EM Counting Module and BDL-488-SMN laser (Becker & Hickl) with a pulse duration of 50 ps and wavelength of 488 nm, and with a CW power equivalent of ∼0.5 mW, externally triggered at a 1 MHz repetition rate. photoluminescence emission from the samples passed through a long-pass optical filter with an edge at 900 nm.

## Additional information

**How to cite this article:** Bodnarchuk, M. I. *et al.* Host–guest chemistry for tuning colloidal solubility, self-organization and photoconductivity of inorganic-capped nanocrystals. *Nat. Commun.*6:10142 doi: 10.1038/ncomms10142 (2015).

## Supplementary Material

Supplementary Information Supplementary Figures 1-5 and Supplementary Table 1
