# Supplementary material for: Host–guest chemistry for tuning colloidal solubility, self-organization and photoconductivity of inorganic-capped nanocrystals
Source: Nat Commun. 2015 Dec 9;6:10142. doi: 10.1038/ncomms10142 (PMC4682102; doi:10.1038/ncomms10142)
Supplement: Supplementary Information — Supplementary Figures 1-5 and Supplementary Table 1 [file ncomms10142-s1.pdf]

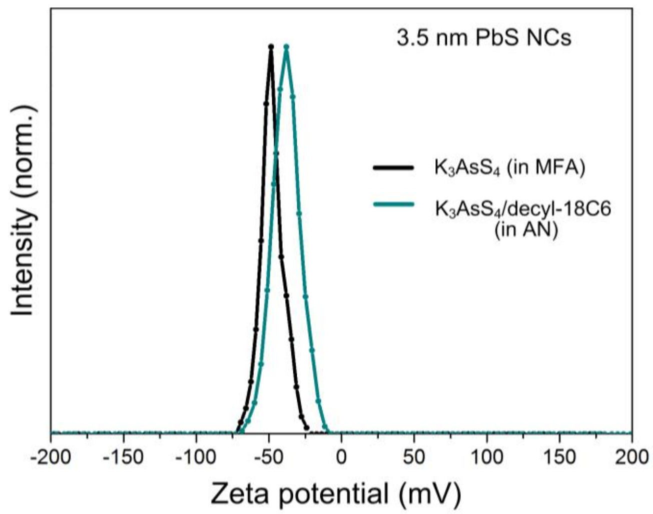

Supplementary Figure 1. Zeta-potentials calculated from electrophoretic mobility measurements presented in Figure 2b.

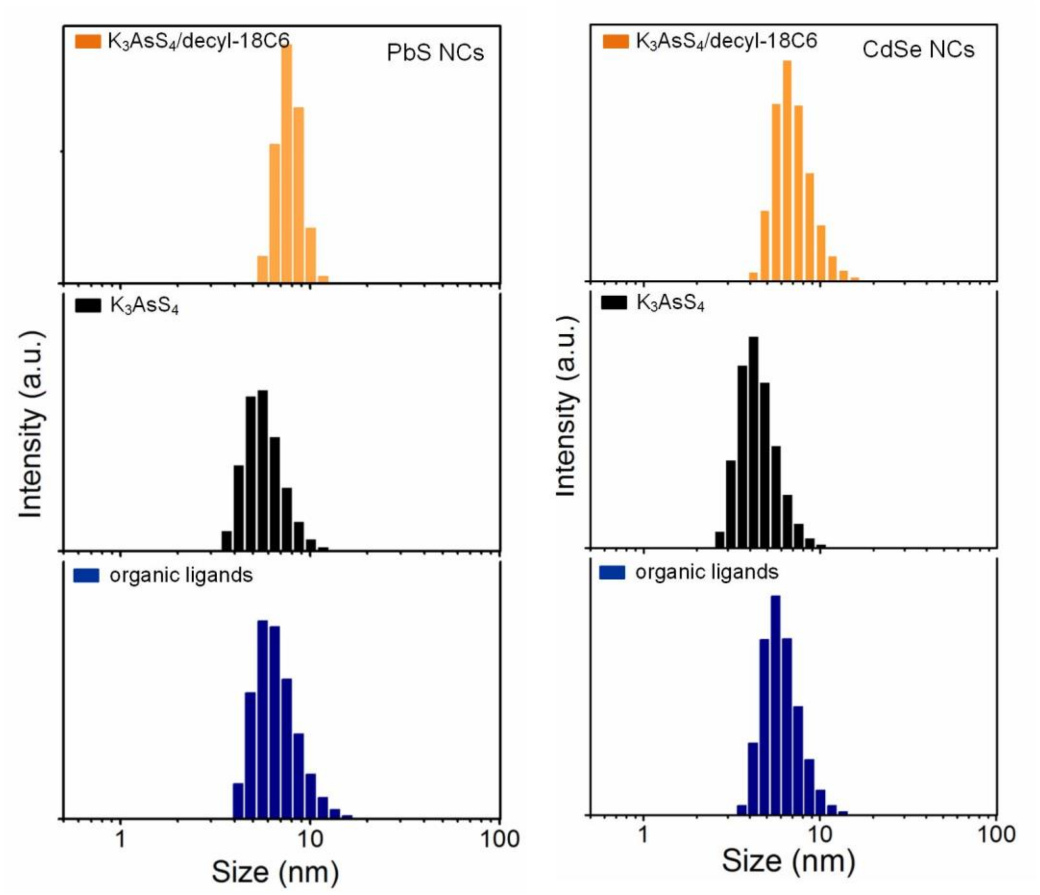

Supplementary Figure 2. DLS measurements of 3.5 nm PbS and 3 nm CdSe NCs with various ligands: oleate (in hexane), K_3AsS_4 (in MFA) and K_3AsS_4-/decyl-18C6 (in DCB).

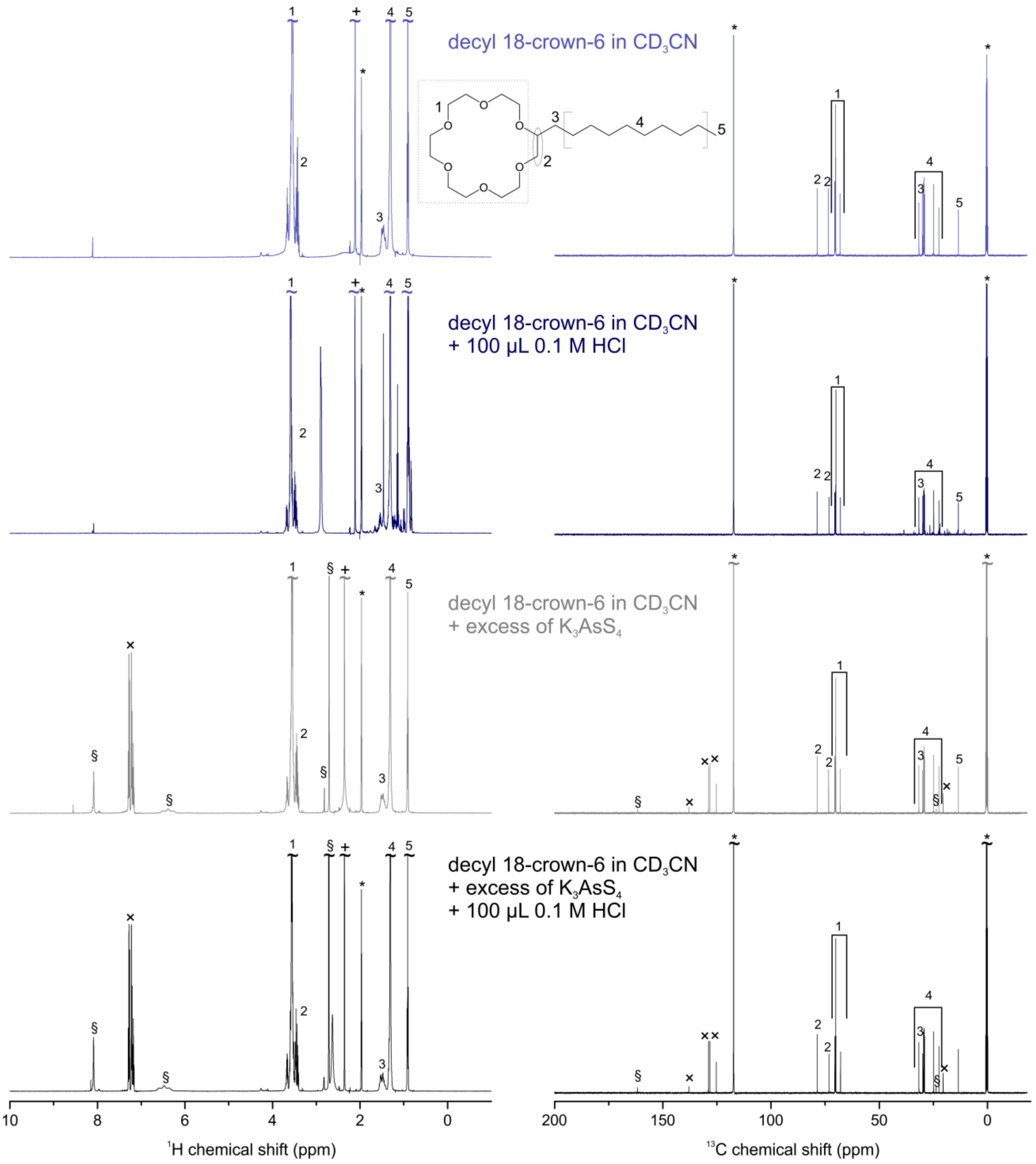

Supplementary Figure 3. ^{1}H and ^{13}C NMR spectra of decyl-18C6 dissolved in CD_3CN, without and with addition of HCl (100 µL of 0.1 M solution). Numbers assign the peaks to the structure of the molecule. Residual solvent peaks from incomplete deuteration or left-overs from sample preparation are signalized by symbols: * (acetonitrile), + (water), ǂ (ethanol), § (N-methylformamide), # (*o*-dichlorobenzene), × (toluene) and Δ (isopropanol).

Supplementary Fig. 3 presents 1-dimensional ^{1}H and ^{13}C NMR experiments with decyl-18C6 dissolved in CD_3CN, without and with the addition of HCl (100 µL of 0.1 M solution), K_3AsS_4

and HCl+K_3AsS_4. These serve as control experiments to test the response of the crown molecule to the solutions identical to those containing PbS NCs and to acids. Clearly, only moderate changes in the chemical shifts of no more than 0.1 ppm can be observed for all proton signals, highlighting the stability of a selected crown derivative towards the components of the solution. ^{13}C NMR spectra are also nearly the same for all four samples. As expected, insertion of K^+ does not lead to substantial changes in the spectra. Peaks were assigned using standard correlation spectra (COSY and HSQC) and are consistent with reported spectra (*J. Phys. Chem.* **1987**, *91*, 3321-3325).

The commonly reported signature for the binding of the organic moieties to the NC surface is significant broadening of all ^1H (and ^{13}C) signals due to either or both (i) slower tumbling of the molecule, approaching the slow dynamics of the whole NC and (ii) binding dynamics, *i.e.* rate of adsorption and desorption, falling in the frequency range of the NMR experiment. A clear test is provided by comparing the NC-crown system with liberated crown. The latter case is obtained here by treating the NC-crown system with an acid HCl destroying the NC. Supplementary Fig. 4 shows that the crown-related peaks in the spectra of PbS/K_3AsS_4/decyl-18C6 are significantly broadened. Addition of an acid, that decolors the colloid due to etching of metal chalcogenide NCs and ligands, fully liberates the crown molecules, as seen from sharp and intense signals, identical to those presented in Supplementary Fig. 3. This result signifies that in the studied colloid there is essentially no “free” crown, and all crown molecules are engaged in dynamic binding.

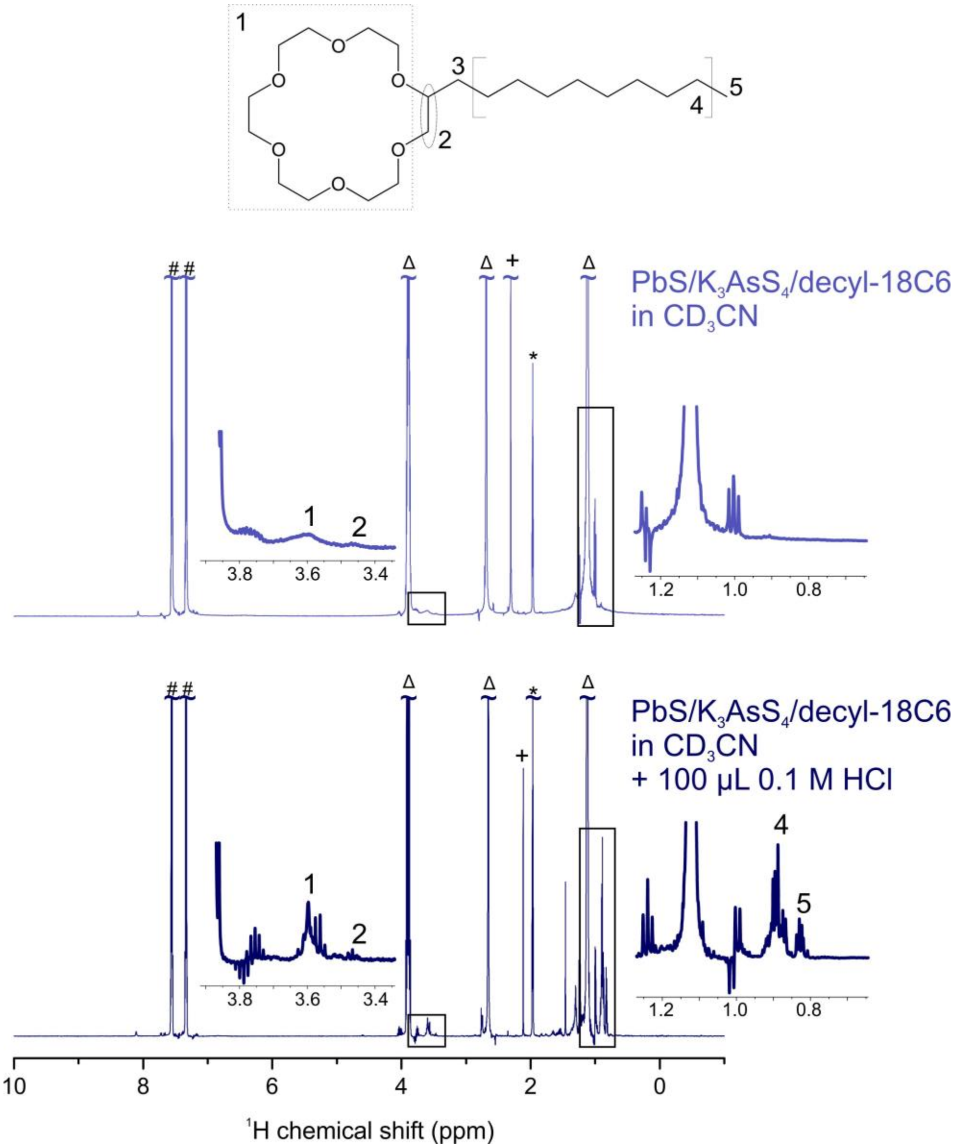

Supplementary Figure 4. ^{1}H NMR study of the binding of decyl-18C6 to the surface of PbS/K_3AsS_4 NCs. Insets carry the main result - appearance of the crown signal upon addition of an acid. Numbers assign the peaks to the structure of the molecule. Residual solvent peaks from incomplete deuteration or left-overs from sample preparation are marked with symbols:
* (acetonitrile), + (water), ǂ (ethanol), § (N-methylformamide), # (*o*-dichlorobenzene), × (toluene) and Δ (isopropanol).

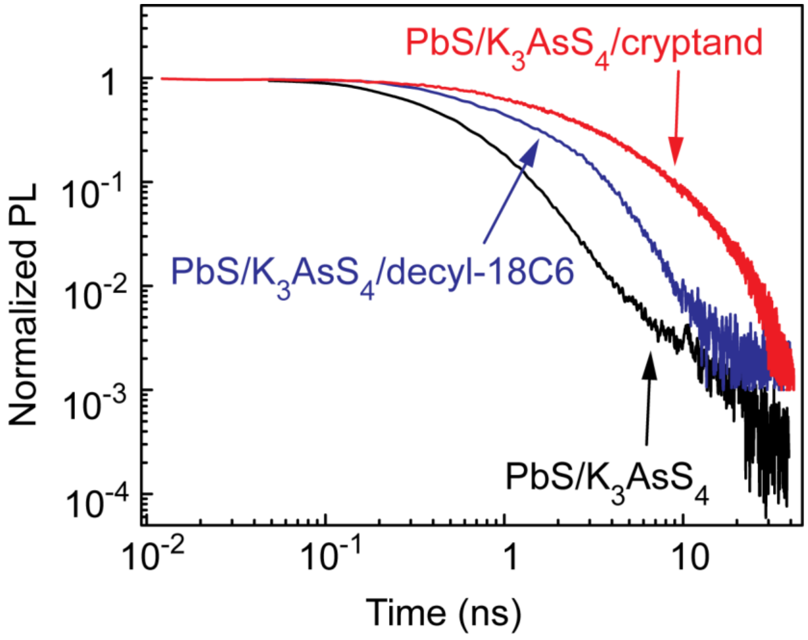

Supplementary Figure 5.Time-resolved photoluminescence decays from films of PbS/K_3AsS_4 NCs with and without the addition of macrocycles. Additional shortening of decay times for macrocycle-free samples may originate from electronic coupling between NCs.

Supplementary Table 1. Elemental analysis by RBS.

3nm PbS NCs	Pb	S	As	K
PbS-oleate	1	0.56 ± 0.03	-	-
PbS/K_3AsS_4	1	1.12 ± 0.05	0.13 ± 0.02	0.33 ± 0.03
PbS/K_3AsS_4/ decyl-18C6	1	0.8 ± 0.3	0.15 ± 0.04	0.4 ± 0.2
